# Two Improvements to the Intentional Stance Theory: Hutto and Satne on Naturalizing Content

**DOI:** 10.1007/s11406-015-9627-1

**Published:** 2015-12-08

**Authors:** Marc Slors

**Affiliations:** Faculty of Philosophy, Theology and Religious Studies, Radboud University Nijmegen, Nijmegen, The Netherlands

**Keywords:** Intentional stance, Mental content, Dennett, Neo-behaviorism, Cultural evolution, Mental states, Intentionality

## Abstract

In this paper I assess the extent to which Daniel Dennett’s Intentional Stance Theory fits into the overall proposal for a programme on naturalizing mental content outlined by Daniel Hutto and Glenda Satne in this issue (Philosophia 43, 2015). I argue that in order to fit the proposal, two changes need to be made: (1) the reality of intentional states should not (just) be grounded in the reality of behavioral patterns but in the ascription-independent status of Ur-intentionality that is the at the root of all intentionality, including content-involving intentonality. This is tricky since (i) Ur-intentionality resembles ‘original intentionality’, which is a notion Dennett rejects, and (ii) the ascription-dependent status of content-involving intentionality should be kept intact. (2) adopting the intentional stance is possible only as part of socio-cultural practices, which implies that this is an exclusively human capacity. I also argue that both changes to the theory are feasible and should be considered improvements relative to the original position developed by Dennett.

In “The Natural Origins of Content (2015)”, Dan Hutto and Glenda Satne (H & S from here on) propose a new, exciting but complex and sketchy research program aimed at a broadly naturalistic understanding of what it means to say that our thoughts, hopes, and beliefs have content. This program is based on a diagnosis of the current state of affairs in philosophical accounts of naturalistic theories of content. Starting with an update of Haugeland’s 1990 review of the ‘Intentionality All Stars’, H & S review the prospects of neo-Cartesian, neo-behaviorist and neo-pragmatist theories of content, finding all of these approaches wanting. The program of naturalizing content they propose as an alternative rests on two pillars. On the one hand they argue that aspects of all three approaches—neo-Cartesianism, neo-behaviorism and neo-pragmatism—must be combined. This joining of forces is made possible, on the other hand, by the real innovation proposed in the paper, a distinction between two kinds of intentionality: ‘ur-intentionality’ and ‘content-involving intentionality’.

For the three strands of theory about content to collaborate, and for these strands to incorporate the distinction between the two kinds of intentionality, it is obvious that some more or less serious modifications to them have to be made. In order to get a grip on what content is supposed to be on H & S’s proposal, it is vital to be clear on what these modifications are. As I will explain below, neo-behaviorism, in particular Daniel Dennett’s Intentional Stance theory, plays a pivotal role in understanding the nature and ontological status of what H & S call ‘content-involving intentionality’. But what modifications to this theory are implied? This is something about which H & S are less than fully clear. In this paper I will spell out the ways in which Dennett’s intentional stance theory should be modified to fit their scheme. The gain will be twofold. On the one hand, as I will show, this proves to be fruitful a means of interpreting and elaborating on H & S’s proposal. On the other hand it will make explicit two serious improvements to the intentional stance theory that are now only vaguely implied by H & S’s paper.

In the first section will identify three questions pertaining to the intentional stance theory as part of H & S’s proposal, which I shall discuss, in turn, in the next three sections. The first of these is connected with the fact that so-called ‘ur-intentionality’ may appear dangerously close to the notion of ‘original intentionality’. The rejection of that notion is an integral part of the intentional stance theory. I shall argue, however, that ur-intentionality is compatible with the main tenets of the intentional stance theory. This will be important since one of the proposed improvements of the intentional stance theory hinges on ur-intentionality.

The second problem is concerned with H & S’s way of dealing with the threat that neo-behaviorism boils down to ‘mere ascriptivism’—the idea that content-involving states only enjoy an ‘as if’ ontological status. The solution to this problem they imply (rather than state explicitly) hinges on the solid, ascription-independent status of ur-intentionality. The ‘as if’ problem, however, pertains to content-involving intentionality. The key to this solution to this problem, then, is in an explicit account—more explicit than provided by H & S—of the connection between ur-intentionality and content-involving intentionality. By sketching this account I will argue that a new and convincing way of dealing with the ‘mere ascription’ problem is provided that is fundamentally different from Dennett’s own more controversial solution.

The third question is concerned with the characterization of what it is to adopt the intentional stance on H & S’s account. I shall first spell out what adopting the intentional stance consists of on this account. I then argue that there is a crucial difference between their view and Dennett’s in this respect. On H & S’s account the intentional stance can only be adopted by humans, whereas Dennett thinks non-human animals adopt the stance as well. I shall argue that H & S’s view on this issue is to be preferred over Dennett’s, since Dennett cannot explain what is means to adopt the intentional stance without circularity while H & S can.

## Hutto and Satne on Content and Intentionality: Three Questions

H & S start with an update of Haugeland’s 1990 review of the ‘Intentionality All Stars’. Some players have changed, but the three basic classes of theory Haugeland identified remain the same. Let me very briefly go over these classes, indicating where traditional positions go wrong according to “The Natural Origins of Content”, what can be retained of them and how these remainders fit into the overall proposed program of naturalizing content. I shall argue that this account raises three questions with respect to the theory that plays a small but pivotal role in explaining content-involving intentionality, the intentional stance theory.

In Haugeland’s Baseball analogy, first base is occupied by neo-Cartesianism. In 1990 that predominantly meant computational functionalists such as Jerry Fodor, Hartry Field and Zenon Pylyshyn. H & S argue that times have changed significantly. For one thing, Fodor (2013) and Pylyshyn now denounce the Fregean notion of ‘sense’ that played a leading role their original position. But more generally, they note with the help for Godfrey-Smith (2006), the overall enthusiasm for an account of mental representations in term of informational semantics has consistently faded over the years. Presently, the most prestigious brand of neo-Cartesianism (if that label is interpreted loosely enough) is the teleosemantic approach of philosophers such as Fred Dretske, Ruth Millikan and David Papineau. According to this approach content can be explained in terms of Darwinian natural selection. Natural selection can instill a specific responsiveness to specific worldly events in organisms. In Dretske’s terms (Dretske 1988) the bottom line is this: when an internal state of an organism indicates a outside event *X* due to causal co-varience, then that state represents *X* if natural selection (or learning) has wired it to cause the organism to behave in a specific (survival enhancing) way towards *X* because of this indication.

The main problem with this approach is that its notion of representation seems a far cry from truth-evaluable mental content. For an organism to respond to food by eating it is not to represent it *as* being this of that kind of food or even as food, a category to be distinguished form other categories. Succeeding in feeding yourself is not the same thing as getting things right. Thus, it seems better not to speak of representation at all (see also Hutto and Myin 2013, p.18 ff.).

But this doesn’t mean that teleosemantics fails to provide an account of intentionality. Surely the frog that responds to a fly by flicking its tongue at it—because it is so wired by evolution—has a basic purposeful, intentional attitude towards a particular part of its surroundings. This attitude, however, is not content-involving. For it does not involve a characterization of the nature of the part of the frog’s environment it is directed at that could be expressed propositionally. *We* might describe the attitude as ‘that is food’ or something similar, but *the frog* doesn’t possess the relevant contrasting concepts to render the notion ‘food’ informative. Instead of content-involving intentionality, then, H & S introduce the notion of ‘ur-intentionality’ to refer to the kind of intentionality that is captured by evolutionary approaches. Ur-intentionality doesn’t involve content. Therefore the term ‘teleo*semantics*’ is somewhat inappropriate. Instead, following Hutto (2008), the term ‘teleosemiotics’ is proposed. Teleosemiotics is what remains of neo-Cartesianism (if that term is still appropriate) on H & S’s proposal. As to the required modifications to this first class of theories, then, it seems to me the proposal is clear and certainly defensible.

When it comes to content-involving intentionality H & S turn to neo-behaviorism and neo-pragmatism. In order to see how both classes of theory are expected to cooperate, with each other and with ur-intentionality, it is best to discuss them together. Schematically, the picture is this: Neo-Cartesianism accounts for ur-intentionality in terms of teleosemiotics. Neo-behaviorism accounts for content-involving intentionality. And Neo-pragmatism explains the connection between the two. Let me first outline how these explanations are supposed to run and then highlight three important questions pertaining to the required modifications to neo-behaviorism.

According to neo-behaviorists, most prominently Daniel Dennett, content-involving intentional states are states of people (organisms, systems) that are aptly described in terms of the beliefs and desires they have. Aptness is not a subjective qualification here, but should be glossed in terms of predictive and interpretive merits. Formulated somewhat more in line with H & S’s terminology, the aptness of a description of a some person’s state in terms of the beliefs and desires she harbours depends on its fitting our *practices* of mental state ascription. Thus, content-involving intentional states such as beliefs and desires are not to be sought at the sub-personal level of the neural mechanisms that underpin our behavior (as neo-Cartesians would have it); they are states of whole persons that figure in people’s reasons for actions, either as self-ascribed or as ascribed by others.

Neo-behaviorism—Dennett’s theory—is controversial. There are two problems in particular with the view that are frequently stated as objections against it. H & S diverge from Dennett exactly on these issues by invoking the help of neo-Cartesianism and neo-pragmatism. In doing so they leave a number of important questions unanswered, however, or so I shall argue.

A first serious problem surrounding Dennett’s position is this. Dennett stresses that beliefs and desires can only be discerned by looking at behavior through the lens of what he calls ‘the intentional stance’. But he is less than fully clear on what this lens consists of (see “The Intentional Stance as a Socio-cultural Achievement” section below; see also Slors 1996). H & S point out that whatever adopting the intentional stance is, explaining how we can adopt it *must* involve explaining how some of us (content ascribers) can wield content-involving intentional concepts. This means that the intentional stance theory can only count as a naturalistic theory of content if it is combined with a naturalistic explanation of how we have come to acquire concepts of content-involving intentional states.

For such an explanation H & S turn to a modified form of neo-pragmatism. From a neo-pragmatists point of view, concepts of content-involving intentional states (beliefs, desires, etc.) are part and parcel of human socio-cultural practices. The meaning of such concepts is in the way we use them. Hence, explaining how we acquired such concepts, in a broadly naturalistic fashion, is explaining how such practices evolved. In principle (though as yet perhaps not in practice) such an explanation can be provided by drawing on two sources. One is the fact that we can start with ur-intentionality. The other is a growing literature on cultural evolution. If we can explain, starting from a situation in which hominids merely display ur-intentionality, how cultural evolution has allowed us to bootstrap ourselves into socio-cultural practices that gradually involve concepts of content-involving intentional states, we have solved the problem of explaining how those who adopt the intentional stance have content-involving intentional concepts at their disposal. This gives us a precondition for adopting the intentional stance.

But does this provide us with an answer to the question what it *is* to adopt the intentonal stance; what the ‘lens’ of the intentional stance consists in? Explaning a precondition for something is not yet to explain the thing entirely. The question is what, on H & S’s position, adopting the intentional stance entails.

A second problem facing neo-behaviorism is how to avoid what H & S call ‘mere ascriptivism’. The problem here is how to avoid the suggestion that neo-behaviorism boils down to claiming that content-involving intentional states are merely ‘in the eye of the beholder’. After all, if content-involving intentional states can only be discerned through the lens of the intentional stance, why not say that they *exist* only from the viewpoint of the onlooker? Some of Dennett’s best and most important writings address this issue (see in particular Dennett 1987, pp. 69–82, and Dennett 1991). According to him the lens of the intentional stance helps us to track and explanatorily exploit patterns in behavior that are ‘out there’. H & S do not discuss this literature. Instead they point to the fact that many remain unconvinced. This does indeed seem to be the case. There is an ongoing discussion over what ‘patterns’ are and in what sense they are ‘real’. And this fact alone shows that these terms cannot do the trick of convincing skeptics that neo-behaviorism is more than mere ascriptivism.

This provides room for a different solution to the problem that is implicitly present in H & S’s paper. On this solution, an ‘as if’ reading of the neo-behaviorist position can be avoided by pointing to the fact that we only ascribe content on the basis of behavior that already displays ur-intentionality. Ur-intentionality, explained in terms of teleosemiotics, is a perfectly objective, ascription independent phenomenon. If content-involving intentionality is an extension of it, then it seems content-involving intentionality is sufficiently rooted in objectivity to avoid an ‘as if’ objection to neo-behaviorism.

But here two further questions arise. A first question that is raised by the strategy to employ ur-intentionality to avoid an ‘as if’ reading of the neo-behaviorist approach to content-involving intentionality is this: The worry about ‘mere ascriptivism’ or ‘as if’ intentionality concerns content-involving intentionality. The objectivity of ur-intentionality can only help to allay worries about content-involving intentionality if the latter is sufficiently rooted in the former. But what does that mean? How and to what extent is ur-intentionality a ‘component of’ ascribed content-involving intentional states?

Secondly, it is not immediately clear how the notion of ur-intentionality would fit in the overall theoretical framework of the intentional stance theory. From the viewpoint of the idea that beliefs and desires can only be discerned through the lens of the intentional stance, it is best to reject, as Dennett does, a strict distinction between literal and metaphorical ascriptions of intentionality. Likewise, it is best to reject the Searlean distinction between original and derived intentionality (Searle 1983, 1992). But ur-intentionality looks a lot like original or literal intentionality. So an important open question raised by H & S’s paper is how the very notion of ur-intentionality can fit into a neo-behaviorist outlook on content and intentionality.

To summarize, H & S’s proposal leaves three questions unanswered with respect to the required modifications to neo-behaviorism: (1) What is it, on H & S’s account to ‘adopt the intentional stance’ or to be an ascriber of content-involving intentional states? (2) What is the connection between ur-intentional states and content-involving intentional states, such that the objectivity of ur-intentionality can save content-involving intentionality from a mere ‘as if’ status? (3) How can the notion of ur-intentionality be fitted into a roughly neo-behaviorist outlook on content? I will discuss these questions, in reverse order, in the next three sections.

## Ur-intentionality is Not ‘Literal’ or ‘Original’ Intentionality

Is the idea of a naturally evolved content-less intentionality, a basic directedness or behavioral purposefulness that exists objectively, independent of ascription compatible with Dennett’s intentional stance theory? Dennett rejects the ideas of ‘literal’ intentionality’ (as opposed to ‘merely metaphorical’ intentionality) or ‘original intentionality’ (to be distinguished from ‘derived’ intentionality). The point of this rejection is to emphasize that all intentionality is ascribed, hence ascribed intentionality is not ‘second-rate’. Thus at first glance it seems the idea of ur-intentionality is incompatible with the basic outlook of the intentional stance theory. I shall argue that this first impression is not correct.

It is not easy to compare Dennett’s views (which sometimes change subtly throughout his oeuvre) with H & S’s. Obviously Dennett does not make the distinction between ur- and content-involving intentionality. When he speaks of intentionality this is at the level of beliefs and desires. But belief and desire ascription do not, on his view, require (linguistic) concept possession, as H & S suggest. According to Dennett, non-human animals can ascribe beliefs and desires just as well as we can. Intentional state ascription, according to him, is not merely a human affair but occurs in many other mammals as well as in some birds (Dennett 1996). It seems Dennett’s intentional stance encompasses anything from full blown ascription of complex linguistically mediated beliefs to what Apperly and Butterfill call ‘registerings’ (Butterfill and Apperly 2013), the most minimal sense in which organisms can be said to have belief-like states.

At these various ‘levels’ Dennett is clear that intentionality is ascribed. This does not exclude the idea that something objective answers to such ascriptions. Behavior to which intentionality is correctly or productively ascribed displays patterns that can only be tracked through intentional state ascription. But the point is that this correctness or productivity does not require the ascribed intentional states to correspond to anything identifiable at the level of the (neural) mechanisms that give rise to the behavior at issue. In that sense intentional states *as such* cannot exist unascribed.

Ur-intentionality does exist unascribed—that is part of the concept. It is the product of natural selection, not of explanatory or predictive strategies. Moreover, ur-intentionality can be explained at the level of neural mechanisms as is illustrated by the (very) brief summary of Dretske’s position above. But, and this is crucial, the twist that H & S give to this idea is that the neural mechanisms behind this unascribed intentionality are content-less. This is *not* yet the intentionality of beliefs and desires, despite what Dretske c.s. claim. Hence, ur-intentionality should not be compared with the intentionality of the intentional stance.

Dretske’s or Papineau’s views are incompatible with Dennett’s because they aspire to provide the story behind the neural underpinnings of the kind of intentionality that according to Dennett we ascribe through adopting the intentional stance. Once that idea is dropped—once teleosemantics is turned into teleosemiotics—it is not so obvious that there is incompatibility. For one thing, Dennett’s views are thoroughly congenial with the Darwinian outlook and he has always emphasized his admiration for and congeniality with Millikan’s views (see e.g., Dennett 1995, 2013). Moreover, in his (2006) he argues that the intentional stance does have an evolutionary precursor that tracks something more basic than what he calls intentionality: agency. Dennett:

More mobile animals have evolved more discriminating methods; in particular, they tend to have the ability to divide detected motion into the banal (the rustling of the leaves, the swaying of the seaweed) and the potentially vital: the *“animate* motion” (or “biological motion”) of another *agent*, another animal with a mind, who might be a predator, or a prey, or a mate, or a rival conspecific. This makes economic sense, of course. If you startle at every motion you detect, you’ll never find supper, and if you don’t startle at the dangerous motions, you’ll soon be somebody else’s supper. (Dennett 2006, 109)

Agency detection (derived from Baron-Cohen’s 1995 notion) is not adopting the intentional stance. The kind of agency involved is strikingly similar to the basic directedness or contentless purposefulness of H & S’s ur-intentionality. And just like H & S claim that content-involving intentionality is built on the basis of ur-intentionality, Dennett describes the intentional stance as “a *further* good trick” (2006, 109; emphasis mine) to not only

distinguish the animate movers from the rest, but draw distinctions between the likely sorts of motions to anticipate from the animate ones: will it attack me or flee, will it move left or right, will it back down if I threaten, does it see me yet, does it want to eat me or would it prefer to go after my neighbor? (Dennett 2006, 109)

Here we may argue over whether or not some of the intentionality that Dennett describes as being covered by the intentional stance would fall in H & S’s realm of ur-intentionality. But the point here is not so much where to draw the line between agency detection and adopting the intentional stance, or between ur-intentionality and content-involving intentionality. The point is that what is tracked through agency detection—what Dennett calls animate or biological motion—is just as much ascription-independent as ur-intentionality is according to H & S. Agency is detected, intentionality is ascribed. Detection is a purely epistemic notion, whereas ascription involves further interpretation.

Thus, while there certainly are differences between Dennett’s views and those of H & S, probably involving the question of where to draw the line between epistemology and hermeneutics when it comes to discerning various forms of intentionality, the very idea of ur-intentionality is not incompatible with Dennett’s outlook. For on the one hand, his intentional stance theory is aimed solely at content-involving intentionality. On the other hand, he does acknowledge a precursor of (content-involving) intentional state ascription, which is the detection of something strikingly close to ur-intentionality.

## Real Content as Socio-culturally Harnessed ur-Intentionality

The next question to be tackled is how ur-intentionality and content-involving intentionality are coupled. The question is pertinent in view of the fact that the ascription-independent character of ur-intentionality can possibly be used to avoid ‘mere ascriptivism’. The point is that ‘mere ascriptivism’ is a charge that is leveled against neo-behaviorist views on content-involving intentionality, such as the intentional stance theory. Hence if ur-intentionality is to help against this charge, there must be a strong connection between ur-intentionality and content-involving intentionality. But what connection, exactly?

Explaining this is a delicate balancing act. For on the one hand the connection must be strong enough to dispel the mere ascriptivism charge. On the other hand, however, the connection cannot be overly strong. For in that case the distinction between ur-intentionality as an ascription-independent phenomenon and content-involving intentionality fades. And that would undermine the idea—central to H & S’s proposal—that content-involving intentionality can be explained in neo-behaviorist terms as an ascription-dependent phenomenon.

The link between ur-intentionality and content-involving intentionality on H & S’s proposal is provided by a combination of neo-pragmatism and theories of cultural evolution. Neo-pragmatism explains the presence of content-involving intentionality in terms of socio-cultural practices people are engaged in. The difference between the frog’s attitude towards the flies that hover around it and my attitude to what’s on my plate in a fancy restaurant is that my responses to the food in front of me, unlike those of the frog, are dependent on various other contextual factors and thoroughly culturally determined. Unlike the frog, I have beliefs and desires about my food. The expression of these beliefs and desires have various (socio-culturally determined) consequences and these consequences co-determine the very meaning of my beliefs and desires—their content.

The trajectory from ur-intentionality to content-involving intentionality can be explained in terms of the evolution of culture. Current literature on that topic focuses mostly on cultural transmission of skills, knowledge and habits (e.g., Sterelny 2012) and on the typically human ability for joint intentionality (e.g., Tomasello 1999). A full explanation of the evolution of culture should also include the various ways in which culture helps to extend human cognition (e.g., Clark 2007), the ways in which human cognitive activities become socially distributed (e.g., Hutchins 1995) and the ways in which humans found ways to develop complex divisions of cognitive labour (Baumeister 2010, 29–30). For the sake of the argument we may assume that a full explanation of the emergence of culture is possible, at least in principle. For in order to answer the question how ur-intentionality is related to content-involving intentionality it suffices to note that on H & S’s account cultural evolution starts from a situation in which hominids are related to each other and to the world in ways determined by ur-intentionality. The question we must answer is whether, how and to what extent the ur-intentionality of this evolutionary starting position is retained in full blown socio-cultural forms of life.

The answer to this question is in H & S’s brief and somewhat cryptic reference to Jesse Prinz’s characterization of teleosemantics. Prinz (2004, 54) aptly describes the core of teleosemantics (or teleosemiotics for that matter) as the idea that intentionality ensues from the fact that organisms are “set up to be set off” by something. An organism’s being set off by *x* (eating *x*, fleeing from *x*, etc.) counts as an intentional response to it only if that organism is set-up to respond in that way to *x* in virtue of the beneficial effects of that response as the result of learning or of natural selection. H & S now describe the processes of cultural transmission of skills, knowledge, habits, rules of conduct etc. as follows: “the mechanism of social conformity that gets the practice of learning and teaching off the ground can be understood as a mechanism to be *set up by others* and to *set up others*.”

The point here is not merely that apart from individual learning and evolution there is a third way for an organism to be “set up to be set off” so as to be intentionally related to its environment (i.e., learning from another organism). The point is also (and more importantly) that the class of things one becomes responsive to through “being set up by others” is dramatically enlarged. Others can make one responsive not merely to physical items in one’s environment and to the basic actions of fellow humans, but also to the rich and specific behavioral repertoire of enculturated human beings. This ranges from simple rules of conduct to the use of language. Likewise, being set-up by others makes one responsive to a humanly appropriated physical world of artifacts, ranging from tools, household objects and buildings to symbols and written language. Being appropriately responsive to the fine-grained differences in the behavior of others, verbal and otherwise, and being appropriately responsive to the world of artifacts is, on a neo-pragmatic understanding, exactly what is required to be endowed with beliefs and desires, i.e., content-involving intentionality.

The transition from being set-up by individual learning or evolution to being set-up by others and setting up others is immense. It is the transition from early hominid cognition to modern human cognition—from nature to culture. And yet: the cognitive *mechanism* at work in the natural phenomenon of ur-intentionality (being set-up to be set-off by something) is the same as the mechanism that is used in the content-involving intentionality of enculturated human beings. Hence, content-involving intentionality is not merely a product of a process of enculturation that ‘starts from’ or is ‘built on top of’ the basic phenomenon of ur-intentionality. Rather, content-involving intentionality is ‘socio-culturally harnessed’ ur-intentionality. Paradoxically, then, despite the *immense* differences between animal cognition and human cognition, the mechanism of ur-intentionality is still *at the heart of* enculturated content-involving intentionality.

And this explains why the ascription-independent reality of ur-intentionality dispels worries about mere ascriptivism in the case of content-involving intentionality.

## The Intentional Stance as a Socio-cultural Achievement

So what remains of the neo-behaviorist idea that content-involving intentional states are essentially ascribed? If content-involving intentionality becomes possible through socio-culturally harnessing ur-intentionality, and if ur-intentionality is ascription independent, why, then, should we think of beliefs and desires as ascription dependent? What does that even mean?

The answer to this question is that while beliefs and desires, as such, are parts of the characterizations, predictions and explanations of a range of actions, it would be a mistake to see them as part of the mechanisms that cause them. These mechanisms are a combination of being set-up—naturally and socio-culturally—to be set off in very specific ways by an immense range of stimuli and the actual stimuli provided by the physical and social environments of people. We may be able to understand and taxonomize the complex ways in which we are set up and hence the ways in which we respond to our natural and social environments by ascribing beliefs and desires. But that doesn’t mean (pace e.g., Dretske 1988) that my believing that there is food on my plate or that I ought to shake hands with the person opposite me is a discrete, identifiable, causally active part of the mechanisms that drive my actions. Beliefs, desires and other propositional attitudes *as such* are characterizations of the ways in which we are responsive to our environments. And characterizations, however unambiguous and inescapable, are ascription-dependent.

But then what is it to be able to provide such characterizations? What is it to be able to adopt the intentional stance? Briefly put, on the picture sketched so far it is being able to be engaged in a specific form of life—ours—and play a specific kind of language game—the language game of folk-psychology. Being able to play this language game depends on being able to track the specific responsiveness of people to their natural, and socio-cultural environments. It consists of classifying and labeling these ways in terms of propositional attitudes such as beliefs, desires, hopes, fears, etc.

It is important to stress that this is an ability that doesn’t come automatically with the practices of setting each other up to be set off by specific inputs. H & S distinguish between intentional patients and intentional agents. The former are beings who play their parts in the socio-cultural game of setting each other up to be responsive to various natural and socio-cultural inputs, i.e., beings whose behavior can appropriately be described in terms of beliefs and desires. The latter are beings who are capable of describing the behavior of intentional patients in terms of beliefs and desires. The point is that it seems in principle possible to be engaged in practices of setting each other up to be set off in specific ways without being able to characterize the ensuing behavior in folk-psychological terms. This is exactly the situation from which Sellars’ Myth of Jones starts (Sellars 1956). The idea behind that fictitious story is to explain the emergence of the ascription of beliefs and desires from a situation in which our ‘ancestors’ merely recognized behavioral patterns and behavioral dispositions. Ascribing beliefs and desires allowed these ‘ancestors’ to better exploit and predict these.

The distinction between being engaged in practices of setting each other up and practices of content-involving intentional state ascription is mirrored by the distinction between intentional patients and intentional agents. But it should be stressed that though this distinction is conceptually apt, it is in fact somewhat artificial. That is, it is hard to imagine a stage in our cultural evolution in which people are engaged in the former practice without being engaged in the latter. This in line with H & S’s warning that overplaying the similarity between intentional patients and intentional agents leads to blurring the distinction between ur-intentionality and content-involving intentionality, while overplaying the difference between patients and agents leads to restricting intentionality to intentional agents only.

Intentional state ascription, adopting the intentional stance, is, on the picture sketched so far, a socio-cultural affair. To ascribe beliefs and desires implicitly or to master the concepts of ‘belief’ and ‘desire’ explicitly, requires being acquainted with human forms of life and the intentionalistic language game of folk-psychology (which may be rather varied across cultures and times, see Lillard 1998, Macdonald 2003, 2007). Here H & S diverge form Dennett. For if the intentional stance is a socio-cultural achievement, the implication would be that only humans can adopt it. As indicated above, by contrast, Dennett seems to think that animals adopt the intentional stance as well as we do.

So here the neo-pragmatist twist that H & S’s program necessarily gives to neo-behaviorism leads to a significant difference with Dennett’s original views. Is this a reason to doubt that H & S can actually incorporate the intentional stance theory in their overall program? I think not. In fact the very notion of an ‘intentional stance’ is somewhat mysterious on Dennett’s use of it while the idea of participating in an intentional language game that in turn is intertwined with practices of setting each other up is not. Let me finish by elaborating on this point and consequently defending (my interpretation of) the proposal by H & S (this is an abbreviated version of the argument in Slors 1996).

It is crucial to keep in mind that the core of Dennett’s intentional stance proposal is that “while belief is a perfectly objective phenomenon (…), it can be discerned *only* from the point of view of one who adopts a certain predictive strategy, and its existence can be confirmed *only* by an assessment of the success of the strategy (…)” (Dennett 1987, p. 14; emphasis mine). This means that there is no use for concepts such as ‘belief’ and ‘desire’ outside of the adopted intentional stance. We only understand these concepts because we are able to adopt the intentional stance.

The consequence of this is that we cannot use these concepts in an explanation of what it means to adopt the intentional stance on pain of vicious circularity. We can discern beliefs and desires only through the lens of the intentional stance. When someone asks what this lens consists of, it is uninformative to answer that it is what allows us to discern beliefs and desires. We knew that. What we want to know is what it is that allows us to do this. Hence, we need an explanation of what it is to adopt the intentional stance that does not presuppose the concepts of belief and desire, but that explains how these concepts are produced by it.

How can we provide such an explanation? It is certainly insufficient to say that adopting the intentional stance is a means of predicting and/or explaining behavior. There are ways of doing that that do not fall under the intentional stance. It seems that if we want to explain what it is to adopt the intentional stance without invoking an intentionalistic vocabulary in our explanans, we must describe what it is that people *do* when they adopt the intentional stance. H & S’s program allows exactly that. In principle it should be possible to provide an accurate description of what people do when they are engaged in socio-cultural routines of ‘setting each other up’, and in the practices of wielding their folk-psychological language to describe these routines. It should be possible to describe when and how words such as ‘belief’ and ‘desire’ are used in these practices. No doubt such a description may be unbelievably difficult to produce in practice. It may consist of descriptions of social rules, of analyses of terms to such as e.g., the differences in direction of fit between beliefs and desires and of elaborate inferential role semantics. But even if we do not know exactly what the explanation is going to look like, there is nothing mysterious about the idea of such an explanation. This is the consequence of conceiving of the intentional stance as a socio-cultural achievement.

Such an explanation is not open to Dennett. On the one hand he seems keen on ascribing the ability to adopt the intentional stance to non-human animals. On the other hand, he insists that it is possible to adopt the intentional stance towards systems that do not play such a part in our forms of life. H & S mention the macromolecule, but the thermostat is perhaps the best known example. Hence, Dennett divorces the notion of the intentional stance from our actual forms of life. And this robs him of the opportunity to explain, non-circularly, what it is that we *do* when we adopt the intentional stance. This gives the intentional stance something of a mysterious air that is absent in H & S’s version of it.

## Summary and Conclusion

I have tried to show that H & S’s appropriation of Dennett’s intentional stance theory is feasible and results in a more plausible and defensible version of the theory. I first argued that the notion of ‘ur-intentionality’ is compatible with the general tenets of the neo-behavioristic outlook. This is crucial, since it functions in at least one of the two improvements to the theory that H & S’s proposal implies. This is the proposal of an elegant new solution to the threat of ‘mere ascritivism’. If, as I argued, we view content-involving intentionality as socio-culturally harnessed ur-intentionality, then the objective reality of ur-intentionality precludes an ‘as if’ reading of content-involving intentional states, even though the latter are ascription-dependent. The second improvement follows from the fact that adopting the intentional stance, on H & S’s proposal, can be explained as being engaged in our intentional form of life and playing the intentional language game of our ‘folk-psychology’. This limits the ability to adopt the stance to Humans. But it allows for the possibility—not open to Dennett—to explain what it is to adopt the intentional stance non-circularly by explaining what it is that people *do* when they adopt the stance.
